# A systematic review and quality analysis of pediatric traumatic brain injury clinical practice guidelines

**DOI:** 10.1371/journal.pone.0201550

**Published:** 2018-08-02

**Authors:** Roselyn Appenteng, Taylor Nelp, Jihad Abdelgadir, Nelly Weledji, Michael Haglund, Emily Smith, Oscar Obiga, Francis M. Sakita, Edson A. Miguel, Carolina M. Vissoci, Henry Rice, Joao Ricardo Nickenig Vissoci, Catherine Staton

**Affiliations:** 1 Duke University School of Medicine, Durham, North Carolina, United States of America; 2 Division of Emergency Medicine, Duke University Medical Center, Durham, North Carolina, United States of America; 3 Division of Global Neurosurgery and Neurology, Department of Neurosurgery, Duke University Medical Center, Durham, North Carolina, United States of America; 4 University of North Carolina School of Medicine, Chapel Hill, North Carolina, United States of America; 5 Duke Global Health Institute, Duke University, Durham, North Carolina, United States of America; 6 Mbarara Regional Referral Hospital, Mbarara, Uganda; 7 Kilimanjaro Christian Medical Center, Moshi, Tanzania; 8 Division of Pediatric Intensive Care, State University of Maringá, Maringá, Paraná, Brazil; 9 Department of General Surgery, North Wing Regional Hospital, Asa Norte, Brasilia, Brazil; 10 Duke Division of Pediatric Surgery, Duke University Medical Center, Durham, North Carolina, United States of America; National Center for Child Health and Development, JAPAN

## Abstract

**Background:**

Traumatic brain injuries (TBI) are a significant cause of mortality and morbidity for children globally. Adherence to evidence-based treatment guidelines have been shown to improve TBI outcomes. To inform the creation of a pediatric TBI management guideline for a low and middle income country context, we assessed the quality of available clinical practice guidelines (CPGs) for the acute management pediatric TBI.

**Methods:**

Articles were identified and retrieved from MEDLINE, EMBASE, Cochrane Library, LILACS, Africa-Wide Information and Global Index Medicus. These articles were screened by four reviewers independently. Based on the eligibility criteria, with the exception of literature reviews, opinion papers and editor’s letters, articles published from 1995 to November 11, 2016 which covered clinical recommendations, clinical practice or treatment guidelines for the acute management of pediatric TBI (within 24 hours) were included for review. A reference and citation analysis was performed. Seven independent reviewers from low, middle and high income clinical settings with knowledge of pediatric TBI management appraised the guidelines using the AGREE II instrument. Scores for the CPGs were aggregated by domain and overall assessment was determined.

**Results:**

We screened 2372 articles of which 17 were retained for data extraction and guideline appraisal. Except for one CPG from a middle income country, the majority (16/17) of the guidelines were developed in high income countries. Seven guidelines were developed specifically for the pediatric population, while the remaining CPGs addressed the acute management of TBI in both adult and pediatric populations. The New Zealand Guideline Group (NZGG, 2006) received the highest overall assessment score of 46/49 (93.88%) followed by the Scandinavian Neurotrauma Committee (SNC, 2016) with a score of 45/49 (91.84%) followed by the Scottish Intercollegiate Guideline Network (SIGN, 2009) and Brain Trauma Foundation (BTF 2012) both with scores of 44/49 (89.80%). CPGs from Cincinnati Children’s Hospital (CCH 2006) and Sao Paulo Medical School Hospital/Brazilian Society of Neurosurgery (USP/BSN, 2001) received the lowest score of 27/49 (55.10%) subsequently followed by the Appropriateness Criteria (ACR, 2015) with 29/49 (59.18%). The domains for scope and purpose and clarity of presentation received the highest scores across the CPGs, while applicability and editorial independence domains had the lowest scores with a wider variability in score range for rigor of development and stakeholder involvement.

**Conclusions:**

To our knowledge, this is the first systematic review and guideline appraisal for pediatric CPGs concerning the acute management of TBI. Targeted guideline creation specific to the pediatric population has the potential to improve the quality of acute TBI CPGs. Furthermore, it is crucial to address the applicability of a guideline to translate the CPG from a published manuscript into clinically relevant local practice tools and for resource limited practice settings.

## Introduction

Traumatic brain injuries (TBI) are a significant cause of mortality and morbidity among children. [1–3] Between 2002 to 2006, for children between 0 to 14, emergency department visits for fall-related TBIs increased by 62% from 290 to 470.5 per 100,000. Children now account for the highest rates of TBI-related emergency room visits across the general population. [1, 4–6]

Although the incidence of pediatric TBI varies broadly, the burden of trauma and associated TBI is comparatively higher in low and middle income countries (LMIC). [2, 7, 8] Globally, 95% of injuries in the pediatric population occurs in LMICs. An estimated 950,000 deaths occur annually due to injuries sustained by children of which 90% are unintentional. Worldwide, injuries are the leading cause of death for children aged 10 to 19 years. In children under 15 years, TBIs account for the highest rates of unintentional injuries. [9, 10] The CRASH trial, a multinational randomized controlled trial evaluating corticosteroid used to treat significant head injury, demonstrated a 2 times higher odds of mortality following severe TBI in LMICs in comparison high income countries (HICs). [8]

While the mechanism of injury varies by age, motor-vehicle crashes are the predominant cause of injury followed by falls, in the US and worldwide. [1, 2, 11] The burgeoning development of motorization and transportation systems remains unmatched by the existing safety infrastructure in low and middle income countries. As such, children have an increased vulnerability to traumatic injuries and TBIs within this context especially as pedestrians. [9, 12] Moreover, the sequelae of TBI extends beyond mortality to the potential for disability and associated emotional and financial cost. [13, 14]

To improve TBI outcomes for the LMIC pediatric population, evidence-based treatment guidelines must serve a pivotal role alongside the continuum of care which integrates injury prevention initiatives with a robust trauma system and comprehensive rehabilitation services. In 1995, the Brain Trauma Foundation developed and subsequently published the *Guidelines for the Management of Severe Traumatic Brain Injury*.[15] The fourth edition continues to focus on identifying the best evidence to guide clinical practice and improve TBI outcomes in the adult population.[16] The *Guidelines for Medical Management of Severe TBI for infants*, *children and adolescents* was later developed in 2003 and a subsequent edition was released in 2012 to synthesize the best evidence and inform clinical practice in the pediatric population.[17,18]

Adherence to guidelines have been shown to improve outcomes in the management of TBIs. Keris et al. demonstrated a reduction in hospital case fatality rate in adult TBI patients with the implementation of TBI guidelines in Latvia.[19] In a retrospective and prospective data analysis, Palmer et al demonstrated an odds ratio of 9.13(95% CI 3.25, 25. 67) of a good outcome in comparison to a poor outcome or death when American Association of Neurological Surgeons Guidelines for the management of severe head injury were adapted for a TBI protocol in community hospital setting.[20] Similar studies have demonstrated improvement in clinical outcomes following the implementation of the Brain Trauma Foundation’s guidelines in both the adult and pediatric population[21–23].

However, implementing a suitable clinical practice guideline (CPG) for the management of acute TBI in an LMIC poses a challenge. Literature on TBI guideline creation and use in LMIC is limited. [24] Of the 24 TBI clinical practice guidelines appraised in a previous systematic review, Patel et al found only one developed in a LMIC. [25] Furthermore, the gap between the available resources and the requisite resource capacity necessary to effect guideline recommendations as well as the variability in trauma systems often remain unaddressed. [25, 26] In order to inform the locally driven creation of acute pediatric TBI management guideline for an LMIC context, we aim to assess the quality of available CPGs for the acute diagnostics and management of pediatric TBI by undertaking a systematic review of the available literature.

## Materials and methods

### Protocol and registration

A protocol for this review is undergoing review for registration in the PROSPERO (International Prospective Register of Systematic Reviews) database (S1 Text). This systematic review is reported according to the Preferred Reporting Items for Systematic Review and Meta-Analyses (PRISMA) Statement (S2 Table). [27]

### Eligibility criteria

The inclusion criteria for articles were the following: abstracts must mention either (clinical) recommendations, (clinical) practice guidelines or treatment guidelines for the acute management of TBI (within 24 hours). Subsequently, full texts were retrieved and excluded if the texts were literature reviews, opinion papers or editor’s letters, published prior to 1995. Furthermore, the clinical practice guidelines (CPGs) had to include the pediatric population as defined by the age range from birth to 18 years or a subset of the pediatric population. The newest versions of the CPGs were included for review, if multiple editions were available. All levels of TBI severity were included in this review. We contacted authors to find English versions of candidate abstracts with published non-English full texts.

### Identification of studies

The search strategy was used to identify relevant abstracts from MEDLINE, EMBASE, Cochrane Library, LILACS, Africa-Wide Information and Global Index Medicus is provided in S1 Table. Additional abstracts were retrieved from the Duke University Medical Center Guidelines repository. These guidelines are sourced from the National Guidelines Clearinghouse, American College of Emergency Medicine, Canadian Medical Association-Clinical Practice Guidelines and National Institute of Health and Clinical Excellence Guidelines. After the full text review, we performed a reference and citation analysis primarily using Web of Science. The citation analysis was augmented with Google Scholar and a manual search of references was performed when necessary.

### Literature search

We identified candidate abstracts with “Craniocerebral Trauma”, “Brain injury”, “Practice Guideline” [Publication Type], “Evidence-Based Medicine” as MeSH terms. Limits to exclude animals, editorials, letters, case reports and comments were also applied in the search strategy as demonstrated in S1 Table for MEDLINE. No language or date limits were applied to the database search.

### Study selection

Four reviewers working in pairs, (R.A. and T.N.) and (J.A. and N.W.) evaluated the titles and abstracts independently. Thereafter, full-text manuscripts were retrieved and independently evaluated with the eligibility criteria for inclusion. Articles included from the reference and citation analysis were also evaluated based on the eligibility criteria. We included articles based on consensus and any disagreements were resolved by a third reviewer (C.S.)

### Data extraction

The two pairs of reviewers, (R.A. and T.N.) and (J.A. and N.W.), independently extracted general characteristics of the CPGs. The information obtained included the following: year of publication, year updated (if multiple versions available), country of guideline development, institution or organization responsible for guideline development and the type of group responsible for the development of the guideline, specific descriptors including professional, academic, non-profit, international or mixed were provided. Additionally, the focus of the guideline whether prehospital care, early management, imaging, ICU or covering the complete spectrum of care was also indicated. The patient population, if present and severity of brain injury reported in the guideline was also extracted. We noted the income delineation of the country or countries of origin for the guideline as high-, middle- or low- income with according to the World Bank’s determination. [28]

### Quality assessment

Seven appraisers from low, middle and high income clinical settings with knowledge of pediatric TBI management independently evaluated the quality of the final CPGs using the Appraisal of Guidelines for Research and Evaluation (AGREE) II instrument. [29, 30] The appraisers were from the USA, Brazil, Tanzania and Uganda. Both English and Portuguese translations of the AGREE II user’s manual were provided to the appraisers. The CPGs were evaluated using an electronic form adapted from the user’s manual. The AGREE II instrument is the current gold standard tool for assessing the quality of CPGs and the quality of the development of the guideline. [31] The tool consists of six thematic domains which are addressed using 23 items. Each item is graded on a seven-point scale with “strongly agree” garnering the highest score of 7 and “strongly disagree” associated with a 1. The six domains include the following: scope and purpose, stakeholder involvement, rigor of development, clarity of presentation, applicability and editorial independence. [29]

Domain one focuses on the scope and purpose of the guideline specifically evaluating the overall objective, pertinent health questions and target population covered by guideline. The stakeholder involvement addressed in domain two determines whether relevant professional groups were involved in the development of the guideline, if the CPGs are reflective of the views and preferences of the target population and seeks for a definition of the guideline’s target users. Domain three determines whether the body of evidence leading to the recommendations in the guidelines, was systematically searched and included for review based on clear selection criteria. Furthermore, this domain determines whether the strengths and weaknesses of the body of evidence were assessed, whether the link between recommendations and the evidence was well delineated in the guideline, if the method(s) for formulating the recommendations was clear and if the recommendations reflect an evaluation of pertinent health benefits, risks and side effects. Lastly, domain three assesses the involvement of an external expert review of the guideline before publication and if there is a method to update the guideline. [29]

Domain four which examines the clarity of presentation of the CPGs evaluates whether the recommendations are specific and unequivocal, present different options for management and if the most important recommendations can be easily identified. Domain five delves into the applicability of the CPG. This domain considers the facilitators and barriers that impact the use of the guideline. Additionally, it evaluates the availability of tools and resources to implement the guidelines, information on the resource utilization cost and economic consideration pertaining to guideline as well as the presence of audit criteria. The editorial independence of the guideline is addressed in the sixth domain, where the potential competing interests of the guideline development group and the influence of the funding body’s views are assessed. [29]

Subsequently, a global assessment of the guideline was performed with an overall seven-point scale quality rating. Here, appraisers also determined whether they would recommend the CPG for use by indicating “yes”, “yes with modifications” or “no”. [29]

### Data analysis

The individual scores for the 23 items were summed within each respective domain, according to the AGREE II user’s manual to determine the overall domain scores for each CPG. Afterwards, the individual domain scores are scaled according to the following formula:

$$\frac{\text{Obtained}\;\text{score}\;-\;\text{Minimum}\;\text{possible}\;\text{score}}{\text{Maximum}\;\text{possible}\;\text{score}\;-\;\text{Minimum}\;\text{possible}\;\text{score}}$$

The highest possible scaled score for a domain is equivalent to 100% while 0% equates to the lowest possible scaled score. The percentage scores were calculated for the six domains of each guideline. A descriptive analysis of the CPGs was performed and an assessment for the CPGs according to the respective domains is provided. An intraclass correlation coefficient (ICC) was calculated using R for statistical computing to assess the consistency of appraisers’ evaluations across each domain. A criteria of 0.7 was used an acceptable inter-rater reliability value. [29, 32]

## Results

### Study selection

**Fig 1:**
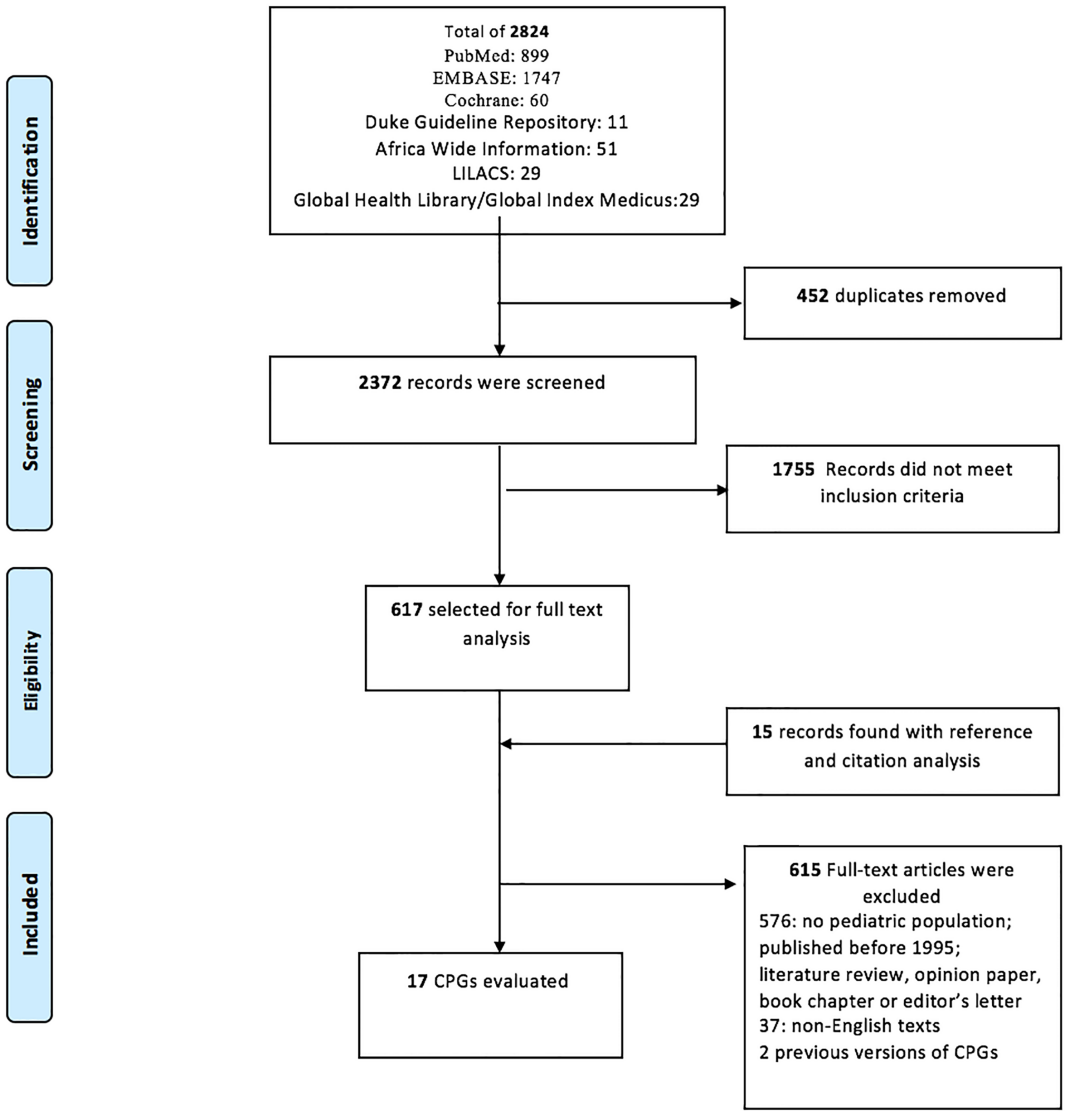
Preferred Reporting Items for Systematic Reviews and Meta-Analyses (PRISMA) Flow Diagram.

The initial search strategy produced 2824 titles and abstracts resulting in 2,372 unique records for review after the removal of 452 duplicates (Fig 1). Subsequently, 1755 abstracts were exempted from further review based on the eligibility criteria. A reference and citation analysis was performed to retrieve an additional 15 manuscripts. Altogether, 632 full texts with CPGs were reviewed according to the eligibility criteria of which 17 were retained for data extraction and guideline appraisal. We contacted authors to find English versions of 37 non-English texts; of the responses we received none that fit inclusion and exclusion criteria were found.

### CPG characteristics

The systematic review of the literature resulted in the appraisal of 17 CPGs (Table 1) for the acute management of pediatric TBI. Although the CPGs originated from 5 different continents namely Asia, South America, Europe and North America, Australia, 10 were developed in North America [18, 33–41]; of these 8 were from USA. [18, 33–35, 37–40] Apart from one guideline [42], all the CPGS were developed in predominantly high income countries or by an international committee representative of a consortium of high income countries. [18, 33–41, 43–48]The 17 guidelines were created by a variety of guideline development groups namely, four professional organizations [33–35, 45], two academic institutions [39,40], three non-profit organizations [18, 36, 37, 48], two national institutes [43, 44], two regional or state trauma programs[38, 41], two international committees [46, 47]and one from a collaboration of an academic and professional institute[42]. The target of 10 of the CPGs included both the adult and pediatric population [18, 34, 37, 38, 42–46, 48] while 7 were restricted primarily to the pediatric population. [18, 33, 35, 36, 39, 40, 47] Altogether, the CPGs spanned the full spectrum of TBI severity. Here, 7 CPGs addressed all levels of severity [33, 38, 41–44, 48] while 6 focused on mild or minor TBI. [34–36, 39, 40, 46] Furthermore, 3 CPGs specifically highlighted the management of severe TBI [18, 37, 45] and a remaining CPG focused on both minor and moderate TBI. [49] The focus of the CPGs varied broadly with one CPG primarily addressing prehospital care [37], seven focused on early management of acute TBI [35, 38, 39, 41, 43, 46, 47] and one CPG [33] specifically addressed imaging in the acute setting. In addition, two guidelines focused on early management and rehabilitation [34, 48], two early management and ICU care [18, 42], two early management and subsequent evaluation or more specifically return to play evaluation in the setting of a concussion. [36, 40] Two guidelines addressed a broader spectrum of care including prehospital care, early management, surgical or specialist care and ICU or neurosurgical unit care. [44, 45]

**Table 1:** Characteristics of clinical practice guidelines.

Guideline Title	Year of publication	Origin	Institution/Guideline development group	Type of Institution or Guideline development group	Focus of the guideline	Patient population	Pediatric age population	Severity of Brain Injury	Country Income status
ACR appropriateness criteria head trauma—Child[33]	2014	USA	American college of Radiology (ACR)	professional organizations	imaging	pediatric	<2 or > or = 2	All levels	HIC
Care of the patient with mild traumatic brain injury [34]	2011	USA	American association of neuroscience nurses (AANN) and Association of rehabilitation nurses (ARN)	professional organizations	early management and rehabilitation	adults and pediatrics	not specified	mild	HIC
Head Injury: Assessment and Early Management[43]	2014	United Kingdom	National Institute for Health and Clinical Excellence (NICE)	national institute	early management	adults and pediatrics	not specified	All levels	HIC
Scottish Intercollegiate guidelines network. Early management of patients with a head injury[44]	2009	Scotland	Sottish Intercollegiate Guidelines Network (SIGN)	National institute	triage, early management, neurosurgery unit	adult and pediatric	not specified	All levels	HIC
Evaluation and management of children younger than two years old with apparently minor head trauma: proposed guidelines[35]	2001	USA	American Academy of Pediatrics (AAP)	Professional organization	Early management	pediatric	<2	Minor (Apparently minor head trauma)	HIC
Guidelines for diagnosing and managing paediatric concussion: Ontario Neurotrauma Foundation guideline[36]	2014	Canada	Ontario Neurotrauma Foundation (ONF)	Non profit organization	early management, and re-evaluation	pediatric	5–18	concussion/mild	HIC
Guidelines for neurosurgical trauma in Brazil[42]	2001	Brazil	Neurosurgical Division of the University of Sao Paolo Medical School Hospital/Brazilian Society of Neurosurgery (USP/BSN)	Academic organization & Professional organization	early management and ICU care	adult and pediatric	not specified	all levels	UMIC
Guide prehospital management of traumatic brain injury 2nd edition[37]	2008	USA	Brain Trauma Foundation (BTF)	Non profit organization	prehospital management	peds and adults	Not specified	severe	HIC
Guidelines for the acute medical management of severe traumatic brain injury in infants, children, and adolescents—second edition[18]	2012	USA	Brain Trauma Foundation (BTF)	Non profit organization	early management and ICU care	pediatric	0–19 (for included studies),	severe	HIC
Guidelines for the management of head injuries in remote and rural Alaska[38]	2003	Alaska, USA	ad hoc committee convened by Alaska Trauma System Review Committee, (ATSRC)18 state-wide physicians, Alaskan Head Trauma Guide Task Force	Ad hoc committee/ state type	early management	adult and pediatric	>2 minimal or >5 for mild some recommendations	all levels	HIC
Guidelines for the Management of Severe Head Injury, 2nd Edition guidelines from the Guidelines Committee on the Management of Severe Head Injury, the Japan Society of Neurotraumatology[45]	2012 (English) 2006-(Japanese)	Japan	Japan Society of Neurotraumatology (JSN)	professional organization	prehospital care, early management, ICU management and surgical management	adult and pediatric	<16	severe	HIC
Mild traumatic brain injury[46]	2012	Europe	European Federation of Neurological Societies (EFNS)	International committee	early management	adult and pediatric	0–5, >5 use adult guidelines	mild	HIC/UMIC
Mild traumatic brain injury in children: just another bump on the head?[39]	2006	USA	Cincinnati Children’s Hospital (CCH)	Academic Institution	Early management	Pediatrics	1 mo—17 yrs	mild	HIC
Mild Traumatic Brain Injury in Children: Practice Guidelines for Emergency Department and Hospitalized Patients[40]	2003	USA	The Departments of Surgery and Pediatrics, the Children’s Hospital of Philadelphia, University of Pennsylvania School of Medicine (CHOP)	Academic Institution	imaging, at home concussion management, return to play	pediatrics	0–18	mild	HIC
Scandinavian guidelines for initial management of minor and moderate head trauma in children[47]	2016	Scandinavia (Norway, Sweden, Denmark, Finland and Iceland)	Scandinavian Neurotrauma Committee (SNC)	International Committee	Early management	Pediatrics	0–18	minor and moderate	HIC
Development of a provincial guideline for the acute assessment and management of adult and pediatric patients with head injuries[41]	2007	Canada	Emergency Health Services (EHS) Nova Scotia Trauma Program	Regional trauma program	early management	adult and pediatric	0–15	all levels	HIC
Traumatic Brain Injury: Diagnosis, Acute Management and Rehabilitation[48]	2006	New Zealand	New Zealand Guideline Group	Non profit organization	Early management and rehabilitation	Adult and pediatric	<17 for imaging; otherwise unspecified	All levels	HIC

### Quality assessment

The AGREE II evaluation of CPGs with the scaled scores and appraisers’ comments are highlighted by domain and the overall assessment is also addressed. The domain specific results are provided in Table 2 while the overall assessment of the CPGs are described in Table 3. The consistency of raters’ appraisal for each domain, which was determined with the interrater reliability is presented in Table 4.

**Table 2:** AGREE II aggregated scores by domain.

CPG	Scope and Purpose Domain 1	Stakeholder involvement Domain 2	Rigor of Development Domain 3	Clarity of Presentation Domain 4	Applicability Domain 5	Editorial Independence Domain 6
ACR, 2014	73.81%	49.21%	44.35%	68.25%	21.43%	52.38%
AANN/ARN, 2011	77.78%	49.21%	66.96%	75.40%	32.14%	33.33%
NICE,2014	92.06%	76.19%	66.07%	88.88%	51.79%	55.95%
SIGN, 2009	92.06%	**89.68%**	**89.58%**	93.65%	**79.17%**	78.57%
AAP, 2001	**97.62%**	70.63%	76.49%	93.65%	50.59%	71.43%
ONF,2014	86.51%	57.94%	47.02%	87.30%	41.67%	39.29%
USP/BSN, 2001	**65.08%**	**35.71%**	**20.24%**	67.46%	**19.64%**	**20.24%**
BTF, 2008	85.71%	59.52%	83.93%	85.71%	39.88%	53.57%
BTF, 2012	91.27%	67.46%	86.90%	92.06%	38.69%	79.76%
ATSRC, 2003	79.37%	50.79%	33.63%	79.37%	32.14%	33.33%
JSN, 2012	85.71%	45.24%	39.29%	**66.67%**	24.40%	32.14%
EFNS, 2012	88.10%	38.88%	55.65%	83.33%	29.17%	63.10%
CCH,2006	78.57%	42.65%	26.49%	70.63%	18.45%	22.62%
CHOP,2003	82.54%	51.59%	36.61%	72.22%	33.93%	39.29%
SNC, 2016	94.44%	66.67%	86.01%	90.48%	51.19%	76.19%
EHS, 2007	87.30%	50%	51.49%	83.33%	33.93%	66.67%
NZZG,2006	92.86%	81.75%	84.23%	**95.24%**	72.02%	**88.10%**

**Table 3:** AGREE II overall guideline assessment.

CPG	Overall Assessment							% of CPG recommendation for use		
	A1	A2	A3	A4	A5	A6	A7	Yes	Mod	No
ACR, 2014	4	6	7	1	5	3	3	42.86%	42.86%	14.29%
AANN/ARN, 2011	2	4	5	5	5	3	6	28.57%	42.86%	28.57%
NICE,2014	5	7	6	6	7	5	6	42.86%	57.14%	0%
SIGN, 2009	5	7	7	6	7	5	7	57.14%	42.86%	0%
AAP, 2001	6	6	6	5	6	6	6	85.71%	14.29%	0%
ONF 2014	6	4	7	5	5	4	5	28.57%	71.43%	0%
USP/BSN, 2001	2	2	4	5	5	3	6	14.29%	42.86%	42.86%
BTF 2008	4	6	6	6	7	5	7	42.86%	57.14%	0%
BTF 2012	6	6	6	7	7	5	7	42.86%	57.14%	0%
ATSRC, 2003	4	5	5	5	2	4	7	28.57%	57.14%	14.29%
JSN, 2012	5	4	6	5	3	6	5	14.29%	71.43%	14.29%
EFNS, 2012	6	5	5	5	6	4	6	42.86%	57.14%	0%
CCH,2006	2	5	4	5	4	2	5	28.57%	28.57%	42.86%
CHOP,2003	5	3	6	4	3	4	5	14.29%	57.14%	28.57%
SNC, 2016	6	7	7	7	6	6	6	85.71%	14.29%	0%
EHS 2007	7	5	3	5	6	5	6	28.57%	57.14%	14.29%
NZGG 2006	6	6	7	7	7	7	6	100%	0%	0%

**Table 4:** Inter-rater reliability for the AGREE II domains.

Domains	ICC	95% CI
Scope and purpose	0.65 (p = 0.00072)*	(0.33,0.86)
Stakeholder involvement	0.78(p = 1.6e-06)*	(0.57, 0.91)
Rigor of Development	0.91(p = 1.4e-15)*	(0.83,0.96)
Clarity of Presentation	0.56(p = 0.0078)*	(0.14,0.82)
Applicability	0.76(p = 4.2e-06)*	(0.54,0.90)
Editorial Independence	0.81(p = 1.1e-07)*	(0.63,0.92)
Overall Guideline Assessment	0.84(p = 1.9e-09)*	(0.69, 0.93)

*Significant to p<0.05, ICC = intra-class correlation coefficient. 95% CI = 95% confidence interval

### Domain one—Scope and purpose

Evaluation and Management of Children Younger than Two Years Old with Apparently Minor Head Trauma: Proposed Guidelines (AAP, 2001) received the highest score of 97.62% in this domain. The lowest score of 65.08% was for the Guidelines for Neurosurgical Trauma in Brazil (USP/BSN, 2001).

### Domain two-Stakeholder involvement

The highest score of 89.68% was from the Early Management of Patients with a Head Injury (SIGN, 2009) while the Guidelines for Neurosurgical Trauma in Brazil (USP/BSN, 2001), with a core of 35.71%, received the lowest score in the domain.

### Domain three-Rigor of development

Guidelines for Neurosurgical Trauma in Brazil (USP/BSN, 2001) received the lowest score of 20.24%. Early Management of Patients with a Head Injury (SIGN, 2009) scored the highest in this domain with 89.58%

### Domain four-Clarity of presentation

Traumatic Brain Injury: Diagnosis, Acute Management and Rehabilitation (NZGG, 2006) received the highest score of 95.24%. Guidelines for the Management of Severe Head Injury, 2nd Edition guidelines from the Guidelines Committee on the Management of Severe Head Injury, the Japan Society of Neurotraumatology (JSN, 2012) received 66.67% as the lowest.

### Domain five-Applicability

Early Management of Patients with a Head Injury (SIGN, 2009) received the highest score of 79.17% in this domain. The lowest score of 18.45% was for mild traumatic brain injury in children: just another bump on the head? (CCH 2006)

### Domain six-Editorial independence

The highest score was 88.10% which was for the Traumatic Brain Injury: Diagnosis, Acute Management and Rehabilitation (NZGG, 2006). Guidelines for Neurosurgical Trauma in Brazil (USP/BSN, 2001) received the lowest score of 20.24%.

### Overall guideline assessment

New Zealand’s Traumatic Brain Injury: Diagnosis, Acute Management and Rehabilitation (NZGG, 2006) received the best overall assessment with 46 out of the maximum 49 points. The Scandinavian guidelines for initial management of minor and moderate head trauma in children (SNC 2016) with 45 out of the maximum 49 points received the next highest overall score. Subsequently, the Early Management of Patients with a Head Injury (SIGN, 2009) and Guidelines for the acute medical management of severe traumatic brain injury in infants, children, and adolescents-second edition (BTF 2012) both received 44 out of 49 points for overall assessment.

At 27 out of 49 points, Mild traumatic brain injury in children: just another bump on the head? (CCH 2006) and Guidelines for Neurosurgical Trauma in Brazil (USP/BSN 2001) received the lowest scores for overall guideline assessment. With a score of 29 out of 49 points, the American College of Radiology’s Appropriateness Criteria Head Trauma—Child (ACR 2014) received the next lowest score.

While the level of recommendation varied for each CPG, all the guidelines were recommended for use. However, Traumatic Brain Injury: Diagnosis, Acute Management and Rehabilitation (NZGG, 2006) received the highest recommendation for use from all appraisers (100%). Subsequently, the Scandinavian guidelines for initial management of minor and moderate head trauma in children (SNC 2016) and the Evaluation and Management of Children Younger than Two Years Old with Apparently Minor Head Trauma: Proposed Guidelines (AAP, 2001) received the next highest recommendation for use; both CPGs were individually recommended by 85.71% of appraisers for use without modification. In contrast, 42.86% of appraisers would not recommend either Mild traumatic brain injury in children: just another bump on the head? (CCH 2003) or the Guidelines for Neurosurgical Trauma in Brazil (USP/BSN 2001) for use.

### Appraisers consistency

Interrater reliability for four domains namely, Stakeholder involvement, Rigor of Development, Applicability, Editorial Independence and Overall Guideline Assessment were above 0.70 demonstrating an acceptable consistency. [50] The domains for Scope and Purpose and Clarity of Presentation, which were lower in consistency, resulted in intraclass correlation values of 0.65 and 0.56 respectively.

## Discussion

To the best of our knowledge, this is the first systematic review and guideline appraisal of CPGs for the acute management of pediatric TBI. Of the 17 CPGs evaluated in this study, ten were developed for both the pediatric and adult population while the remaining guidelines were specifically created for the pediatric population. On average, the domains for scope and purpose and clarity of presentation received the highest scaled scores across the CPGs in contrast to the domains for applicability and editorial independence which received the lowest scores. In this study, the CPGs with the best overall assessments include two guidelines restricted to the pediatric population, namely the Scandinavian guidelines for initial management of minor and moderate head trauma in children (SNC 2016) and the Guidelines for the acute medical management of severe traumatic brain injury in infants, children, and adolescents (BTF 2012). Furthermore, guidelines appraised as the best quality were created by professional guideline development groups with broad expertise and the experience of creating previous versions of these guidelines. Only one CPG was created by a guideline development group from an upper middle income country (UMIC); majority of the guidelines were developed in high income countries (HICs). Our findings suggest that future acute pediatric TBI guidelines may benefit in quality when the guideline is population specific and recognized as an adaptive process with the advantage of being developed with the expertise of a dedicated guideline development body. The missing perspectives of LMICs in the process of guideline creation is essential in TBI care.

The guidelines with the highest overall assessment were developed by the Scandinavian Neurotrauma Committee, Scottish Intercollegiate Guidelines Network, the Brain Trauma Foundation and the now dissolved New Zealand Guideline Group. Moreover, the CPGs, SNC 2016, SIGN 2009, BTF 2012 and NZGG 2006 received the highest scores for rigor of development. This domain evaluates whether the CPG uses a robust systematically searched evidence base that is critically appraised by a development team with broad clinical and technical expertise to directly inform CPG recommendations. The four guidelines shared two overarching factors: not only were the CPGs developed by professional guideline groups, but the members of these groups had clinical, research and methodological expertise well suited to the endeavor and the CPGs with the exception of SNC 2016, had undergone iterations with previous pediatric editions. However, SNC 2016 was borne out of the experience of creating multiple TBI guidelines. [51–53]. As such the process of CPG is best viewed as progressively adaptive and the quality is significantly informed by the composition and expertise of the group creating the guideline. [54]

In this study, two guidelines limited to the pediatric population emerged among the appraised guidelines with the highest overall quality. These CPGs are specifically The Scandinavian guidelines for initial management of minor and moderate head trauma in children (SNC, 2016) and the Guidelines for the acute medical management of severe traumatic brain injury in infants, children, and adolescents (BTF, 2012) [18, 44, 47, 48] In contrast, NZGG 2006, SIGN 2009 and NICE 2007 which address both adult and pediatric TBI received the highest overall assessment and received the highest recommendation for use in previous CPG appraisals.[25, 44, 48, 55, 56] Our findings suggest that the quality of TBI guideline development may significantly benefit from addressing a specific population; Moreover, AAP 2001, a pediatric specific guideline, was appraised as the highest quality CPG in the domain for scope and purpose, the most highly scored domain. Notably, the domain for scope and purpose addresses the central impetus for the development of a guideline: the health questions pertinent to the guideline endeavor, the population of interest and the overall guideline objective. Targeted guideline development around a population serves to streamline evidence and prioritize recommendations, all details that are crucial to improving the quality of the guideline. [25, 55, 56]

The dearth of CPGs from LMICs in the context of the multiple CPG and CPG updates from HIC highlights a missing perspective in TBI guideline development. One of several issues inherent in using CPGs developed in HIC in LMIC is the recommendations may not result in the same outcome. This situation was best demonstrated in the South American trials: Treatment of Intracranial Pressure (BEST Trip) trials where following ICP monitoring recommendations resulted in no significant difference between patients treated by ICP monitoring protocol and patients whose treatment was based on imaging and clinical examination.[57, 58] Current efforts by the Global Neurotrauma Research group are underway to create guidelines for the management of severe TBI for LMIC by determine whether intracranial hypertension in severe TBI patients can be managed without ICP monitoring.[59] Not only must a CPG be adaptable to a local context but the evidence base for recommendations must be interpreted within that context as well. AGREE II indirectly addresses the potential for adaptation as a guideline strategy or direct implementation of a guideline by evaluating the quality of the applicability domain. However, similar to previous TBI CPG appraisals, this was the weakest domain in our review. Improving the quality of CPGs in addressing how well the guidelines translate from published material into actual clinical practice tools can potentially impact the adaptation of CPGs for resource limited settings. The World Health Organization’s Guidelines for Essential Trauma Care has demonstrated efforts to create guidelines that can be adapted across a range of resource settings and along with guideline adaptation tools, good quality TBI CPGs can be adapted in LMICs. [60, 61]

## Limitations

Despite the extensive search of databases with access to both English and non-English texts, it is possible that we may have missed CPGs from countries with unpublished or non-English TBI guidelines. To the best of our efforts, we contacted authors of non-English texts with potential abstracts for English versions of the guidelines. Furthermore, we contacted Chinese medical schools and medical centers with online publications on acute TBI for local CPGs but such guidelines within our scope were unavailable. Additionally, AGREE II guideline is inherently a subjective tool with a potential for bias. However, based on the moderate to high interrater reliability, the increased number of appraisers and diverse clinical experience of the appraisers the potential for bias was minimized significantly.

## Conclusion

CPGs for the acute management of pediatric TBI, as tools for evidence-based medicine, have the capacity to inform the development of trauma care systems and improve the quality of health care delivery. Targeted guideline creation for this specific population has the potential to improve the quality of acute pediatric TBI CPGs. Moreover, considering the guideline development process as adaptive over the long term creates the opportunity to build expertise in guideline development which in turn informs the quality of the CPG. It is crucial to address the applicability of a guideline to translate the CPG from a publication into a clinically relevant local practice tools and for resource limited practice settings.

## Supporting information

S1 Table Search strategy for MEDLINE. (DOCX)

S2 Table Preferred Reporting Items for Systematic Reviews and Meta-Analyses (PRISMA) 2009 checklist. (PDF)

S1 Text PROSPERO registration. (PDF)
