# Supplementary material for: A systematic review and quality analysis of pediatric traumatic brain injury clinical practice guidelines
Source: PLoS One. 2018 Aug 2;13(8):e0201550. doi: 10.1371/journal.pone.0201550 (PMC6072093; doi:10.1371/journal.pone.0201550)
Supplement: S1 Table — (DOCX) [file pone.0201550.s001.docx]

**S1 Table. Search strategy for MEDLINE**

PubMed 10.20.2016

| #1 | “Craniocerebral Trauma”[Mesh:NoExp] OR “Brain Injuries”[Mesh] OR “Head Injuries, Closed”[Mesh] OR “Head Injuries, Penetrating”[Mesh] OR “Intracranial Hemorrhage, Traumatic”[Mesh] OR “Skull Fractures”[Mesh] OR ((“Brain Injuries”[tiab] OR “Brain Injury”[tiab]) AND (Trauma[tiab] OR traumatic[tiab])) OR “Post-Concussive Encephalopathies”[tiab] OR “Post-Concussive Encephalopathy”[tiab] OR “Postconcussive Encephalopathies”[tiab] OR “Postconcussive Encephalopathy”[tiab] OR “Post-Traumatic Encephalopathies”[tiab] OR “Post-Traumatic Encephalopathy”[tiab] OR “Posttraumatic Encephalopathies”[tiab] OR “Posttraumatic Encephalopathy”[tiab] OR “Traumatic Brain”[tiab] OR TBI[tiab] OR “Traumatic Encephalopathy”[tiab] OR “Traumatic Encephalopathies”[tiab] OR “Brain Trauma”[tiab] OR “Brain Traumas”[tiab] OR “intracranial injury”[tiab] OR “intracranial injuries”[tiab] OR “cerebral trauma”[tiab] OR “cerebral traumas”[tiab] | **113,315** |
| --- | --- | --- |
| #2 | “Practice Guideline”[Publication Type] OR “Evidence-Based Medicine”[Mesh] OR “practice guideline”[tiab] OR “practice guidelines”[tiab] OR guideline[ti] OR guidelines[ti] | 136,390 |
| #3 | #1 AND #2 | 1,069 |
| #4 | #3 NOT (Animals[mesh] NOT "Humans"[mesh) | 1,069 |
| #5 | #4 NOT (Editorial[ptyp] OR Letter[ptyp] OR Case Reports[ptyp] OR Comment[ptyp]) | 899 |
