# Supplementary material for: A systematic review and quality analysis of pediatric traumatic brain injury clinical practice guidelines
Source: PLoS One. 2018 Aug 2;13(8):e0201550. doi: 10.1371/journal.pone.0201550 (PMC6072093; doi:10.1371/journal.pone.0201550)
Supplement: S1 Text — (PDF) [file pone.0201550.s003.pdf]

NHS
National Institute for
Health Research

A systematic review and quality analysis of Pediatric Traumatic Brain Injury Clinical Practice Guidelines.

Roselyn Appenteng, Taylor Nelp, Jihad Abdelgadir, Nelly Weledji, Catherine Staton, Joao Vissoci

Citation

Roselyn Appenteng, Taylor Nelp, Jihad Abdelgadir, Nelly Weledji, Catherine Staton, Joao Vissoci. A systematic review and quality analysis of Pediatric Traumatic Brain Injury Clinical Practice Guidelines.. PROSPERO 2017 CRD42017071108 Available from: http://www.crd.york.ac.uk/PROSPERO/display_record.php?ID=CRD42017071108

Review question

What is the quality of the available clinical practice guidelines for the acute management of pediatric traumatic brain injuries?

Searches

We will search the following electronic bibliographic databases: MEDLINE, EMBASE, Cochrane Library, LILACS, Africa-Wide Information and Global Index Medicus and the Duke University Medical Center Guidelines repository which includes guidelines sourced from the National Guidelines Clearinghouse, American College of Emergency Medicine, Canadian Medical Association-Clinical Practice Guidelines and National Institute of Health and Clinical Excellence Guidelines.

The search strategy will include the following MeSH terms “Craniocerebral Trauma”, “Brain injury”, “Practice Guideline” [Publication Type] and “Evidence-Based Medicine” The search terms will be adapted for use with other bibliographic databases There will be no language or date restrictions. A reference and citation analysis will be perfomed using Google Scholar, Web of Science and a manual search of references where appropriate.

Types of study to be included

No restrictions on the types of study design eligible for inclusion

Condition or domain being studied

Management of acute traumatic brain injury.

Participants/population

Children (0-18 years) with acute traumatic brain injury.

Intervention(s), exposure(s)

The Institute of Medicine defines clinical practice guidelines as "statements that include recommendations intended to optimize patient care that are informed by a systematic review of evidence and an assessment of the benefits and harms of alternative care options." The intention of this systematic review is to evaluate the quality of clinical practice guidelines for the management of acute traumatic brain injury in children.

The inclusion criteria for articles were the following: abstracts must mention either (clinical) recommendations, (clinical) practice guidelines or treatment guidelines for the acute management of TBI(within 24 hours). Articles or guidelines must be published between 1995 to November 11th, 2016 and must include the pediatric population or a pediatric subpopulation.

Exclude literature reviews, opinion papers or editor’s letters and articles or guidelines published prior to 1995. Only the newest versions of the CPGs will be included for review, if multiple editions are available. All levels of TBI severity are to be included in this review.

Comparator(s)/control

None

NHS
National Institute for
Health Research

Primary outcome(s)

This review aims to identify, as best as possible, the available published clinical practice guidelines for the management of acute traumatic brain injury in children with the intended goal of assessing the quality of the development of the guidelines.

Secondary outcome(s)

None

Data extraction (selection and coding)

Four reviewers will independently extracted general characteristics of the CPGs. The information obtained included the following: year of publication, year updated (if multiple versions available), country of guideline development and world bank income delineation of the country, the institution or organization responsible for guideline development and the type of group responsible for the development of the guideline, specific descriptors including professional, academic, non-profit, international provided. Additionally, the focus of the guideline whether prehospital care, early management, imaging, ICU care will be extracted. indicated. The patient population,if present and severity of brain injury reported in the guideline will also be extracted. Discrepancies will be resolved by a fifth reviewer

Risk of bias (quality) assessment

The risk of bias will not be assessed in this study but the quality of the included clinical practice guidelines will be assessed using the AGREE II instrument, the current gold standard for assessing the quality of clinical practice guidelines.

Strategy for data synthesis

The data will be summarized descriptively

Analysis of subgroups or subsets

None

Contact details for further information

Roselyn Appenteng
rappenteng@gmail.com

Organisational affiliation of the review

None
None

Review team members and their organisational affiliations

Ms Roselyn Appenteng. Duke School of Medicine
Dr Taylor Nelp. Division of Emergency Medicine, Duke University Medical Center
Dr Jihad Abdelgadir. Division of Global Neurosurgery and Neurology, Department of Neurosurgery, Duke University Medical Center
Ms Nelly Weledji. UNC School of Medicine
Dr Catherine Staton. Division of Emergency Medicine and Division of Global Neurosurgery and Neurology, Duke University Medical Center
Dr Joao Vissoci. Division of Emergency Medicine and Division of Global Neurosurgery and Neurology Duke University Medical Center and and Duke Global Health Institute

Collaborators

Dr Oscar Obiga. Duke Global Health Institute
Dr Francis Sakita. Kilimanjaro Christian Medical Center
Dr Caroline Vissoci. Department of General Surgery, North Wing Regional Hospital
Dr Edson Miguel. Division of Pediatric Intensive Care, State University of Maringá

Anticipated or actual start date

03 October 2016

Anticipated completion date

14 July 2017

Funding sources/sponsors

None

Conflicts of interest

Dr. Staton acknowledges salary support funding from the Fogarty International Center (Staton, K01 TW010000-01A1)

Language

English

Country

United States of America

Stage of review

Ongoing

Subject index terms status

Subject indexing assigned by CRD

Subject index terms

Brain Injuries, Traumatic; Child; Humans; Pediatrics

Date of registration in PROSPERO

03 July 2017

Date of publication of this version

03 July 2017

Stage of review at time of this submission

Stage	Started	Completed
Preliminary searches	Yes	Yes
Piloting of the study selection process	Yes	Yes
Formal screening of search results against eligibility criteria	Yes	Yes
Data extraction	Yes	Yes
Risk of bias (quality) assessment	Yes	No
Data analysis	Yes	No

Versions

03 July 2017

PROSPERO
This information has been provided by the named contact for this review. CRD has accepted this information in good faith and registered the review in PROSPERO. CRD bears no responsibility or liability for the content of this registration record, any associated files or external websites.
